# The Hydroxylated Carbon Nanotubes as the Hole Oxidation System in Electrocatalysis

**DOI:** 10.3390/ma17143532

**Published:** 2024-07-17

**Authors:** Paweł Szroeder, Przemysław Ziółkowski, Ihor Sahalianov, Piotr Madajski, Marek Trzcinski

**Affiliations:** 1 Faculty of Physics, Kazimierz Wielki University, Powstańców Wielkopolskich 2, 85-090 Bydgoszcz, Poland; przemyslaw.ziolkowski@ukw.edu.pl; 2 Laboratory of Organic Electronics, Department of Science and Technology, Linköping University, SE-60174 Norrköping, Sweden; ihor.sahalianov@liu.se; 3 Faculty of Chemistry, Nicolaus Copernicus University, Gagarina 7, 87-100 Toruń, Poland; piotr.madajski@doktorant.umk.pl; 4 Faculty of Chemical Technology and Engineering, Bydgoszcz University of Science and Technology, Kaliskiego 7, 85-796 Bydgoszcz, Poland; marekt@pbs.edu.pl

**Keywords:** carbon nanotubes, hole doping, electron density of states, heterogeneous electron transfer

## Abstract

The hydroxylated carbon nanotubes (CNTs-OH), due to their propensity to trap electrons, are considered in many applications. Despite many case studies, the effect of the electronic structure of the CNT-OH electrode on its oxidation properties has not received in-depth analysis. In the present study, we used Fe(CN)_6_^3−/4−^ and Ru(NH_3_)_6_^3+/2+^ as redox probes, which differ in charge. The CNT-OH and CNT electrodes used in the cyclic voltammetry were in the form of freestanding films. The concentration of holes in the CNTs-OH, estimated from the upshift of the Raman G-feature, was $2.9\times 10^{13}\;{cm}^{-2}$. The standard rate constant of the heterogeneous electron transfer (HET) between Fe(CN)_6_^3−/4−^ and the CNTs-OH electrode was $25.9\times 10^{-4}\;cm\cdot s^{-1}$. The value was more than four times higher than the HET rate on the CNT electrode ($k_{s}=6.3\times 10^{-4}\;cm\cdot s^{-1}$), which proves excellent boosting of the redox reaction by the holes. The opposite effect was observed for the Ru(NH_3_)_6_^3+/2+^ redox couple. While the redox reaction rate constant at the CNT electrode was $1.4\times 10^{-4}\;cm\cdot s^{-1}$, there was a significant suppression of the redox reaction at the CNT-OH electrode ($k_{s}<0.1\times 10^{-4}\;cm\cdot s^{-1}$). Based on the DFT calculations and the Gerischer model, we find that the boosting of the HET from the reduced form of the redox couple to CNT-OH occurs when the reduced forms of the redox couples are negatively charged and the occupied reduced states are aligned with acceptor states of the nanotube electrode.

## 1. Introduction

The unique feature of the carbon nanotubes (CNTs), essentially graphene cylinders, is the symmetry of the bands formed by the carbon bonding π and antibonding π*, orbitals near the charge neutrality point (CNP), which translates into an electron and a hole symmetry in the vicinity of the Fermi level, $\epsilon_{F}$. This symmetry can be easily violated by substitution of carbon atoms in the sp^2^ hexagonal lattice by donor or acceptor dopants [1,2] as well as electrostatic [3,4] or electrochemical gating [5,6]. From the point of view of electron band engineering, chemical functionalization is crucial, since even a slight modification of the π and π* bands significantly affects the electron and hole transport properties and electrocatalytic activity of the nanotubes.

The effect of chemical modification on electronic properties has been explored since 1997, when Rao et al. [7] adapted the methodology for studying the phenomenon of charge transfer between graphene layers and intercalates in graphite intercalated compounds (GIC) to carbon nanotubes. This method takes advantage of the coupling between phonons and free electrons in the graphene (or carbon nanotube) lattice, resulting in a change in the phonon frequencies observed in the Raman spectra [8,9,10]. The interaction between free electrons and phonons at the Fermi level also leads to a broadening of the Raman G-band [8,11].

The hydroxylated carbon nanotubes (CNTs-OH) can be considered donor–acceptor complexes in which the nanotubes play the role of the donor. Consequently, free holes are generated in the nanotube, making it a heterogeneous catalyst that serves as an active oxidative species. As has been shown by Bradley et al. [12], strong hydroxylation using the Fenton reaction does not disrupt the muti-walled carbon nanotube (MWCNT) structure and results in increased surface polarity and higher enthalpy of immersion in water. Deeper insights into the structure, electronic properties, and aggregation behavior of the hydroxylated nanotubes (CNT-OH) have been provided by López-Oyama et al. [13], who have shown that the hydroxylated multi-walled carbon nanotubes (MWCNTs-OH) are partially soluble at the air/water interface, forming extended foam-like carbon networks. The density functional theory (DFT) calculations reported in that work have confirmed that the electron transfer from a nanotube to OH groups results in the formation of the hole carriers in CNT walls. A similar result has been reported in ref. [14].

CNTs-OH, as the hole oxidation system, can be coupled with photocatalysts to enhance the oxidation of pollutants in wastewater, the hydrogen evolution reaction, and the CO_2_ reduction [15,16,17,18]. Low efficiency of the known photocatalysts, such as TiO_2_, halogen oxides, sulfides, and graphite carbon nitride (g-C_3_N_4_), is a consequence of the high recombination rate of photoinduced electron-hole pairs. Zhao et al. [19] has used the CNT-OH/carbon nitride nanobelt composite as a photocatalyst for tetracycline hydrochloride degradation and H_2_ evolution. Due to the high electron trapping ability, enhanced electron transfer from carbon nitride nanobelts to CNTs-OH occurs. As a result, the degradation rate of tetracycline hydrochloride in the CNT-OH/carbon nitride nanobelts system is four times higher than in CNT/carbon nitride nanobelts.

The propensity of CNTs-OH to trap electrons has been exploited in the design of a flexible counter electrode for a dye-sensitized solar cell in which nitrogen-doped graphene/CNT-OH has exhibited superior electrocatalytic performance for the I^−^/I_3_^−^ redox couple [20]. A similar mechanism is used in the electrochemical detection of p-nitrophenol found in industrial wastewater by the neodymium vanadate/CNT-OH composite electrode [21] and detection of antiviral drugs in water by the ionic liquid/CNT-OH electrode [22]. Chemical p-doping by attaching functional groups is the route for fabrication of conducting polymer/CNT composites by electropolymerization [23,24].

Despite many case studies, systematic research on CNTs-OH, considered as a hole oxidation system, lacks an in-depth analysis of electrocatalytic activity, particularly the effect of the electron band structure of modified nanotubes on the kinetics of the heterogeneous electron transfer (HET) reaction. This paper aims to partially fill this gap in research. In this study, we used binder-free CNT-OH and CNT (as a reference) freestanding films (buckypapers) as working electrodes for cyclic voltammetry studies of HET between Fe(CN)_6_^3−/4−^ (negatively charged redox probe) and Ru(NH_3_)_6_^3+/2+^ (positively charged probe) and carbon nanotubes. The CNT and CNT-OH bulk electrodes were examined by Raman spectroscopy to assess the hole and defect concentration. The results are supplemented by the DFT calculations of electron density of states (DOS) that revealed the generation of acceptor states in CNT-OH, which under certain circumstances, i.e., appropriate charge of the reduced form of the redox couple and the alignment of acceptor states of the electrode and the occupied reduced states of the redox couple, boost or quench the oxidation rate.

## 2. Materials and Methods

The pristine and OH-functionalized multi-walled carbon nanotubes (MWCNTs), 5–20 μm in length and 10–30 nm in diameter, were provided by the Institute of Carbon Technologies Llc., Toruń, Poland. According to the technical sheet provided by the supplier, the content of the -OH group in the MWCNTs-OH is 2.8 wt% (~2.0 at%).

The buckypapers were prepared by vacuum filtration of the ultrasonicated nanotube suspensions in 1 wt% aqueous solution of sodium dodecyl sulphate (SDS). The suspensions were filtered on a membrane filter (Millipore ATTP04700, (Merck KGaA, Darmstadt, Germany), pores of 0.8 μm in diameter). The deposited nanotube films were washed with distilled water, dried, and peeled off from the filter.

The electrical measurements were carried out by the four-point method using a Keithley 2450 source measure unit (Textronix China Ltd., Shanghai, China). The Raman spectra were recorded using a Bruker Senterra Raman microscope (Bruker Optics GmbH & Co. KG, Ettlingen, Germany) at an excitation wavelength of 532 nm (2.33 eV) and laser beam power of 20 mW. X-ray photoelectron spectroscopy (XPS) measurements were performed using a VG-Scienta R3000 spectrometer (Scienta Omicron, Uppsala, Sweden) and an Al Kα radiation source (hν = 1486.6 eV). The experimental data were deconvoluted using CasaXPS software (Version 2.3.16) (Casa Software Ltd., Teignmouth, UK). The cyclic voltammetry (CV) studies were performed in a three-electrode configuration using an AUTOLAB PGSTAT204 potentiostat (Metrohm AG, Herisau, Switzerland). Buckypapers, partly dipped in the electrolyte, served as working electrodes. As the redox probes, 5 mM Fe(CN)_6_^3−/4−^ and 1 mM Ru(NH_3_)_6_^3+/2+^ in 0.5 M KCl aqueous solution were used. 

The density functional theory (DFT) was used to investigate the electronic structure of pure and modified nanotubes. The simulation model of a pure nanotube consisted of 420 atoms. We used the ωB97XD [25] exchange-correlation functional and 6-31G(d) basis set, as was approbated before as a reasonable combination to investigate the electronic structure of large organic systems consisting of hundreds of atoms [26,27,28]. Numerical simulations were conducted with the Gaussian16 computational package [29]. The electronic density of states (DOS) of the nanotube was extracted from the Gaussian output data by using the GaussSum software (Version 3.0, https://gausssum.sourceforge.net/) [30].

## 3. Results

### 3.1. The Electrical Conductivity

**Figure 1:**
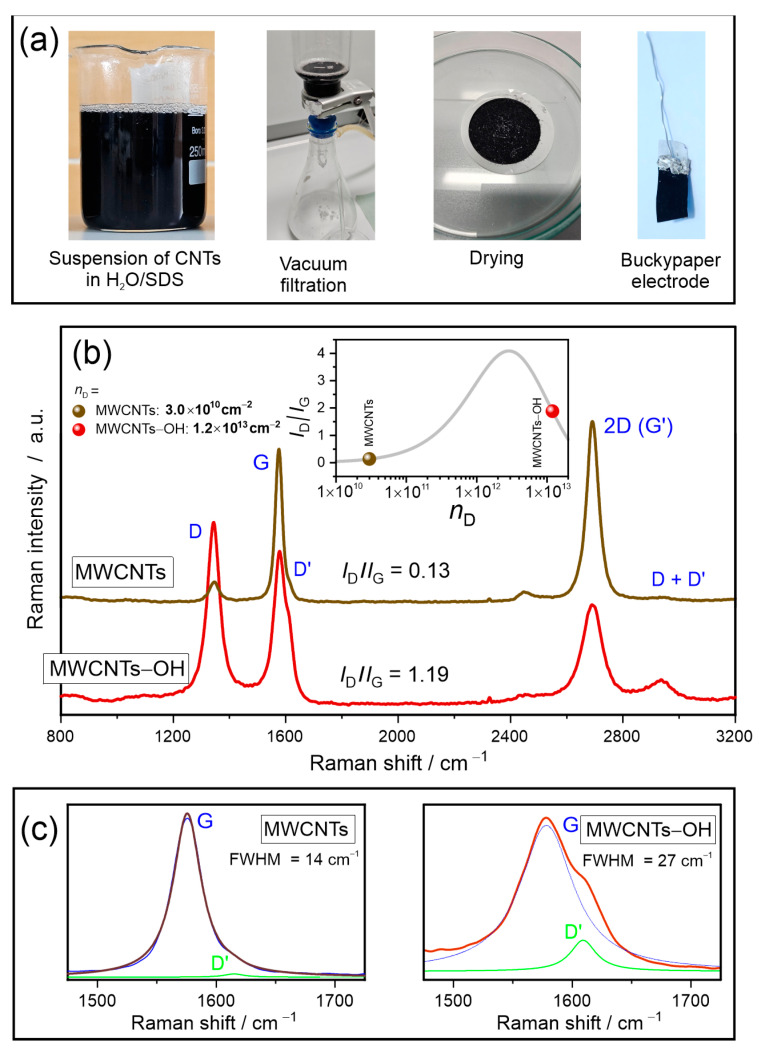
(**a**) The preparation of the buckypaper electrode from the suspension of carbon nanotubes in H_2_O/SDS. (**b**) The Raman spectra of the MWCNT and the MWCNT-OH buckypaper electrodes. The inset shows the dependence of the *I*_D_/*I*_G_ ratio on defect density, *n*_D_. (**c**) A section of the Raman spectrum covering the G and D’ bands along with the results of the peak deconvolution.

Figure 1a shows the procedure of buckypaper preparation. The MWCNT and OH-MWCNT buckypaper films shown in Figure 1 were 480 and 220 μm thick, respectively. The sheet resistances were found to be 170 and 79 mΩ/square, respectively, that correspond to the volume conductivity of $1.2\times 10^{2}\;S\;{cm}^{-1}$ and $5.7\times 10^{2}\;S\;{cm}^{-1}$.

### 3.2. The Raman Spectroscopy

The Raman spectra of the MWCNT and MWCNT-OH buckypapers are shown in Figure 1b. In the spectra, all bands typical of the sp^2^-bonded carbon structures are clearly observed and are labeled according to the nomenclature proposed by Jorio and Saito [31]. The main Raman feature is the G-band at 1576 cm^−1^ formed by the first-order Raman scattering related to the in-plane C-C bond stretching vibrations of a hexagonal carbon lattice. The 2D (G’) band at 2690 cm^−1^ is the second order, the hexagon-breathing mode. Its first-order counterpart, the D line, appears at 1345 cm^−1^ and is Raman inactive in the perfect hexagonal lattice. Other disorder-induced Raman features are the D’ band at 1610 cm^−1^ and the D + D’ combined mode at 2935 cm^−1^. The D’ band partially overlaps with the G band, so in Figure 1c, we have shown a section of the Raman spectrum covering both bands along with the result of the Breit–Wigner–Fano peak function fit. The most important parameters of the main Raman bands, from which information about the outflow of electrons from the hexagonal walls of the nanotubes to the -OH functional groups and the concentration of point defects on the walls can be extracted, are summarized in Table 1.

#### 3.2.1. The Hole Doping

The result of the G-band and D’ band fit (blue and green lines in Figure 1c) shows a clear broadening of the G-band and an increase in the relative intensity of the disorder-induced D’ band. The G-band maximum, $\omega_{G}$, in the MWCNT-OH buckypaper is blue-shifted by 2.7 cm^−1^ relative to the $\omega_{G}$ in pristine MWCNTs (Table 1). In considering the hole doping-induced blue shift of the Raman G-band, we appeal to the previous studies of the charge transfer between carbon CNTs and electron acceptors [7,32]. In that work, the degree of the charge transfer (donor doping) is defined by the quantity of f, which is the transferred charge per carbon atom in the host nanotube. To estimate the value of f in MWCNTs-OH, we used the results of the Raman studies on the charge transfer between the carbon nanotubes and the sulfuric acid from which $\Delta \omega_{G}/\Delta f\approx +350\;{cm}^{-1}$ is obtained [32]. Based on these findings, we conclude that there are $7.7\times 10^{-3}$ holes per carbon atom in MWCNT-OH. Taking the surface density of carbon atoms in graphene to be $3.8\times 10^{15}\;{cm}^{-2}$, we determined the concentration of holes to be $n_{h}=2.9\times 10^{13}\;{cm}^{-2}$.

#### 3.2.2. The Crystallinity

The crystalline quality is reflected by the $I_{2D}/I_{G}$ ratio, which has been found to be 1.18 and 0.65 for MWCNTs and MWCNTs-OH (Table 1). As shown by Santangelo et al. [33], nanotubes with the best graphite wall crystallinity show an $I_{2D}/I_{G}\approx 1.2$. The decrease in the $I_{2D}/I_{G}$ value in MWCNTs-OH indicates a clear deterioration of long-range graphite order.

#### 3.2.3. The Defects

The decrease in the 2D band intensity resulting from the deterioration of crystallinity is correlated with an increase in the defect-activated D-band. Here, it should be pointed out that the intensities of D’ and D+D’ are coupled with the intensity of the D band. Based on the empirical dependence of the $\left(I_{D}/I_{G}\right)$ ratio (inset of the Raman plots) on point defect concentration, $n_{D}$ in cm^−2^, derived for graphene by Lucchese and Cançado [34,35], is given by the following:

(1) $$\frac{I_{D}}{I_{G}}=7.66\times 10^{11}{\cdot \lambda }_{L}^{4}\cdot \left(e^{-9.8696\times 10^{-14}\cdot n_{D}}-e^{-8.4977\times 10^{-13}{\cdot n}_{D}}\right),$$

where $\lambda_{L}$ is the wavelength of the laser excitation light in nm. The relationship (1) is the transformed version of Equation (1) from ref. [35], in which the values of the fit constants for the empirical data are replaced by their numerical values. The $I_{D}/I_{G}$ curve is plotted in the inset of Figure 1b together with the $I_{D}/I_{G}$ values derived from the Raman spectra of the MWCNT and MWCNT-OH buckypapers. From the curve, we find that the defect concentrations in MWCNTs and MWCNTs-OH are 3$.0\times 10^{10}\;{cm}^{-2}$ and 1$.2\times 10^{13}\;{cm}^{-2}$, respectively. The estimated concentration of defects is lower than the concentration of holes, proving that humid air induces additional hole doping, as reported in the ref. [36].

### 3.3. The Surface Chemical Characterization

**Figure 2:**
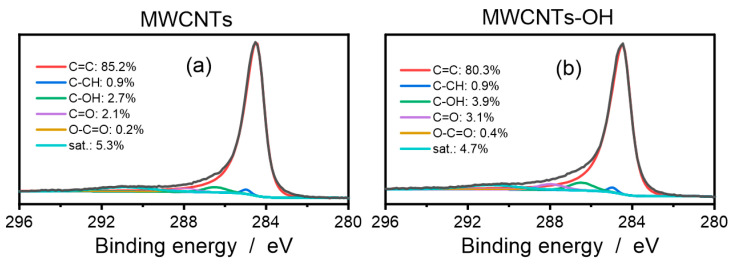
The XPS core Cls spectra of the MWCNT (**a**) and the MWCNT-OH (**b**) buckypapers with the XPS peak area percentages.

The surface chemical characterization of the buckypapers includes the binding states of the carbon delivered XPS study. The atomic percentages of C and O, calculated from the XPS survey spectra, were 96.2 at% and 3.8 at% in the MWCT buckypaper and 93.0 at% and 7.0 at% in the MWCNT-OH buckypaper, respectively. The relatively high oxygen content in the pristine MWCNT buckypaper proves permanent adsorption of water in the interstitial space of the nanotubes forming the buckypaper film [37]. Figure 2 shows the core C1s spectra of the MWCNT and OH-MWCNT buckypapers, which were deconvoluted according to the procedure for carbon nanotubes described by Biesinger [38]. The C1s spectra were separated into six different peaks caused by sp^2^ C=C bonds (284.5 eV), sp^3^ C-CH (285.2 eV), C-OH (286.5 eV), C=O (288 eV), O-C=O (289 eV), and the broad π-π* shake-up satellite peak (290.9 eV). Evidently, the area percentage of the main C=C peak in the MWCNT-OH buckypaper is smaller by 4.9% than the area percentage of the peak in the MWCNT buckypaper. The C-CH peak (area percentage of 0.9% in the MWCNT and MWCNT-OH buckypapers) comes from residues of the SDS. In the C1s spectrum of the MWCNT buckypaper, the C-OH, C=O, and O−C=O sub-peaks are present with a percentage area of 2.7%, 2.1%, and 0.2%, respectively, which proves the surface contamination of the nanotube film with airborne organic compounds [39]. The area percentage of the C-OH and C=O peaks increased by almost a half in the MWCNT-OH buckypaper, which is caused by contribution of OH groups directly bonded to the nanotube walls. It should be emphasized here that the area percentages obtained by deconvolution of the C1s peak should not be directly read as atomic percentages.

### 3.4. The Cyclic Voltammetry

**Figure 3:**
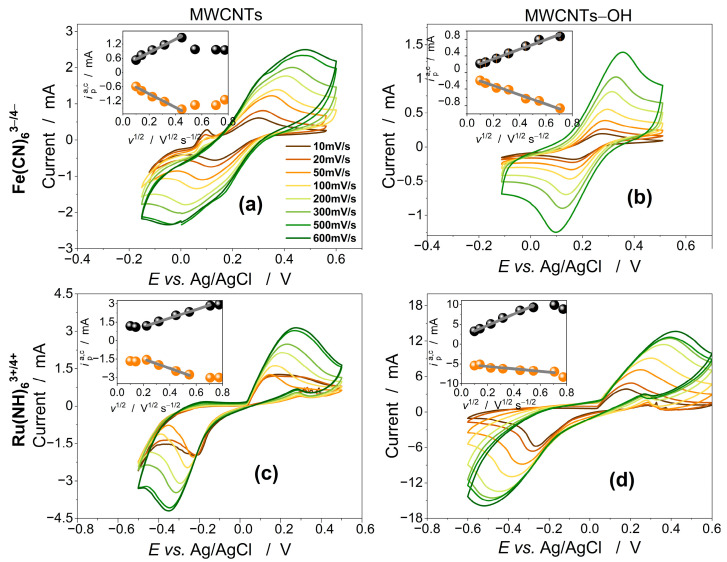
(**a**,**b**) CVs of the 5 mM Fe(CN)_6_^3−/4−^ redox mediator in 0.5 M KCl aqueous solution on the MWCNT and OH-MWCNT buckypaper electrodes, respectively. (**c**,**d**) CVs of the 1 mM Ru(NH_3_)_6_^3+/2+^ redox mediator in the same supporting electrolyte. The insets show dependence of anodic, $i_{p}^{a}$, and cathodic, $i_{p}^{c}$, peak currents toward square root of the sweep rate.

The CVs of the Fe(CN)_6_^3−/4−^ (negatively charged ions) and Ru(NH_3_)_6_^3+/2+^ (positively charged ions) redox couples obtained on the MWCNT and MWCNT-OH buckypaper electrodes are compared in Figure 3. For all applied scan rates (10–600 mV·s^−1^), pairs of anodic and cathodic redox peaks appear in the CVs.

It should be pointed out that redox current peak separation in many of the CVs shown in Figure 3 exceeds 500 mV, above which electrode reactions are considered as non-reversible. Then, the reaction is not controlled solely by diffusion of redox species through the electrolyte/electrode interface. Diffusive-kinetic mixed control and mass transport contribute to the increased peak currents. This phenomenon is most visible for the Ru(NH_3_)_6_^3+/2+^ redox couple on the MWCNT-OH electrode (Figure 3d), where extremely high peak currents are observed along with large peak separation.

#### 3.4.1. The Anodic Charge Transfer Coefficient

In the insets of the CV plots in Figure 3, we show the dependence of the anodic and cathodic peak current, $i_{p}^{a,c}$, on the square root of the scan rate, $v^{1/2}$. The peak currents of the Fe(CN)_6_^3−/4−^ redox mediator on the MWCNT buckypaper electrode are linear at scan rates lower than 200 mV·s^−1^, whereas on the MWCNT-OH electrode, the linearity is maintained over the entire range of scan rates used. The $i_{p}^{a,c}÷v^{1/2}$ dependence of the Ru(NH_3_)_6_^3+/2+^ redox mediator on the MWCNT electrode is linear in the range between 50 mV/s and 500 mV/s (anodic current peak) and between 50 mV/s and 300 mV/s (cathodic current peak). In turn, the linearity range at the MWCNT-OH buckypaper electrode is in the area between 10 mV/s and 300 mV/s (anodic current peak) and between 20 mV/s and 500 mV/s (cathodic reaction peak). To calculate the anodic charge transfer coefficient, $\alpha_{a}$, we used the Laviron equation [40] $\alpha_{a}=\delta_{p}^{a}/(\delta_{p}^{a}-\delta_{p}^{c})$, where $\delta_{p}^{a}$ and $\delta_{p}^{c}$ are the slopes of the anodic and cathodic peak currents vs. log v. We compare the obtained values of $\alpha_{a}$ in Table 2. In both redox media, the anodic charge transfer coefficient is evidently higher on the MWCNT-OH electrode.

#### 3.4.2. The HET Kinetics

The HET kinetics between the Fe(CN)_6_^3−/4^ redox couple and the buckypaper electrodes are manifested by the peak separation, $\Delta E_{p}$, that, at the scan rate of 20 mV/s, is found to be 188 mV and 107 mV at the MWCNT and the MWCNT-OH electrodes, respectively. At the same scan rate, significantly a greater $\Delta E_{p}=369$ and 478 mV, respectively, is observed on the CVs of Ru(NH_3_)_6_^3+/2+^.

We used $\Delta E_{p}$, determined from the CV curves, along with the values of α to estimate the dimensionless Nicholson parameter, ψ, from the following equation [40]:

(2) $$\psi =2.18{\left(\frac{\alpha }{\pi }\right)}^{1/2}\exp\left(-f\alpha^{2}n\Delta E_{p}\right),$$

where $f=F/RT=38.922\;V^{-1}$ at 298.15 K, R is the universal gas constant, and F is the Faraday constant. When the occurring HET is quasi-reversible, the ψ parameter can be converted to the HET standard rate constant, $k_{0}$, using the following formula:

(3) $$\psi =k_{0}Sv^{-1/2}.$$

Here, v is the scan rate in V·s^−1^; $S={\left(\pi nDf\right)}^{-1/2}$, where n is the number of the transferred electrons (n=1); and D is the diffusion coefficient of the redox probe aqueous supporting electrolyte ($D=7.3\times 10^{-6}\;{cm}^{2}\;s^{-1}$ for Fe(CN)_6_^3−/4−^ redox couple [41] and $8.4\times 10^{-6}\;{cm}^{2}\;s^{-1}$ for Ru(NH_3_)_6_^3+/2+^ [42]). The values of coefficient S used in the calculations are $33.471\;{cm}^{-1}\;s^{1/2}\;V^{1/2}$ and $31.202\;{cm}^{-1}\;s^{1/2}\;V^{1/2}$ for Fe(CN)_6_^3−/4−^ and Ru(NH_3_)_6_^3+/2+^, respectively.

**Figure 4:**
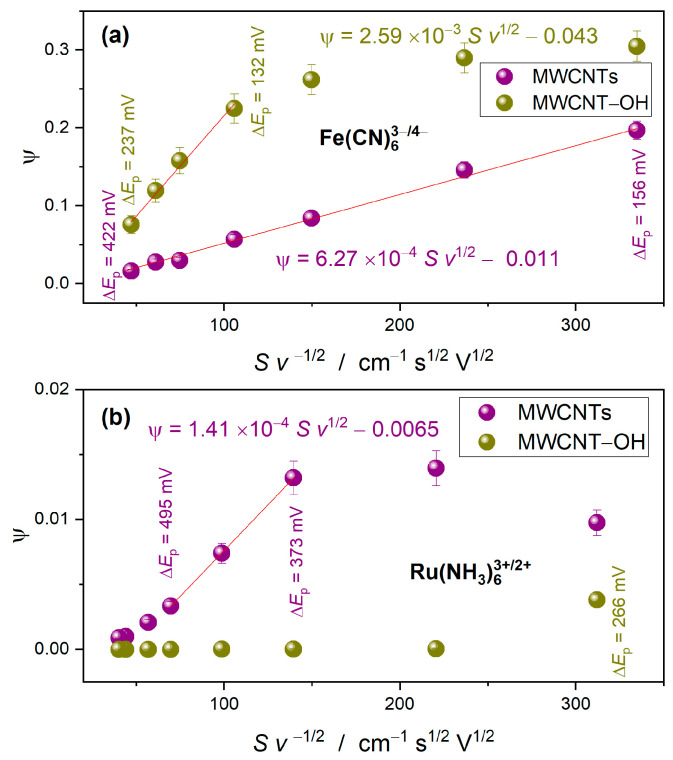
The dependence of the kinetic parameter ψ on $S\cdot v^{1/2}$ on the MWCNT and OH-MWCNT electrodes for Fe(CN)_6_^3−/4−^ (**a**) and Ru(NH_3_)_6_^3+/2+^ (**b**).

The ψ vs. $Sv^{-1/2}$ plots are shown in Figure 4. As linear dependence of ψ occurs for $\Delta E_{p}$ in the interval 130–500 mV, we used the linear parts of the plots for estimation of the HET standard rate constants. As is observed in Figure 4b, extremely low values of ψ show for Ru(NH_3_)_6_^3+/2+^ on the MWCNT-OH buckypaper electrode. For scan rates higher than 20 mV/s, the $\Delta E_{p}$ is larger than 550 mV, and the corresponding ψ are smaller than $10^{-5}$, indicating an irreversible process. For this electrode, we roughly estimated the $k_{0}$ from Equations (2) and (3) from $\Delta E_{p}=266\;mV$ measured at the scan rate of 10 mV/s.

The calculated values of the $k_{0}$ are compared in Table 2. Fundamental differences in electrode reaction kinetics can be observed between the Fe(CN)_6_^3−/4−^ and Ru(NH_3_)_6_^3+/2+^ redox probes. Generally, the faster HET occurs between the negatively charged Fe(CN)_6_^3−/4−^ redox couple and carbon nanotube electrodes. The hole doping induced by sidewall hydroxylation results in further enhancement of the kinetics, which is manifested by a four-fold increase in the $k_{0}$. The $k_{0}$ value of HET between Ru(NH_3_)_6_^3+/2+^ and MWCNTs is more than four times lower than in the case of Fe(CN)_6_^3−/4−^. The hole doping by hydroxylation results in further diminution of $k_{0}$.

These results demonstrate that the charge of the redox mediator and the hole doping of the nanotube electrode by hydroxylation (as is revealed by Raman spectroscopy) play key roles in the HET kinetics. Positively charged Ru(NH_3_)_6_^3+/2+^ ions have a more limited access to the MWCNT electrode than Fe(CN)_6_^3−/4−^ ions, which results in slower kinetics.

The holes induced by nanotube hydroxylation facilitate the oxidation of the reduced forms and hinder the reduction of the oxidized forms of the redox media. The anodic charge transfer coefficient, $\alpha_{a}$, on the MWCNT-OH electrode is greater than 1/2 for both redox media (Table 2). However, while the $\alpha_{a}$ is slightly greater than 0.5 for the negatively charged Fe(CN)_6_^3−/4−^ redox couple, the positively charged Ru(NH_3_)_6_^3+/2+^ couple is much faster oxidized than reduced ($\alpha_{a}=0.737$). Thus, when considering MWCNT-OH as a hole oxidation system in electrocatalysis, the charge of the species being oxidized must be taken into account.

### 3.5. The Electron Density of the States of the CNT-OH Electrode

In order to provide a model that would explain the effect of MWCNT hydroxylation on their electrocatalytic properties, we have calculated the π-electron density of states (DOS) of the metallic SWCNT (5,5). Since the separation between walls in MWCNTs is as small as 0.35 Å, only the outer cylinders of the multi-walled nanotube can be hydroxylated. Thus, the OH-functionalization of the outer tubule of the MWCNT is similar to that of the SWCNT.

**Figure 5:**
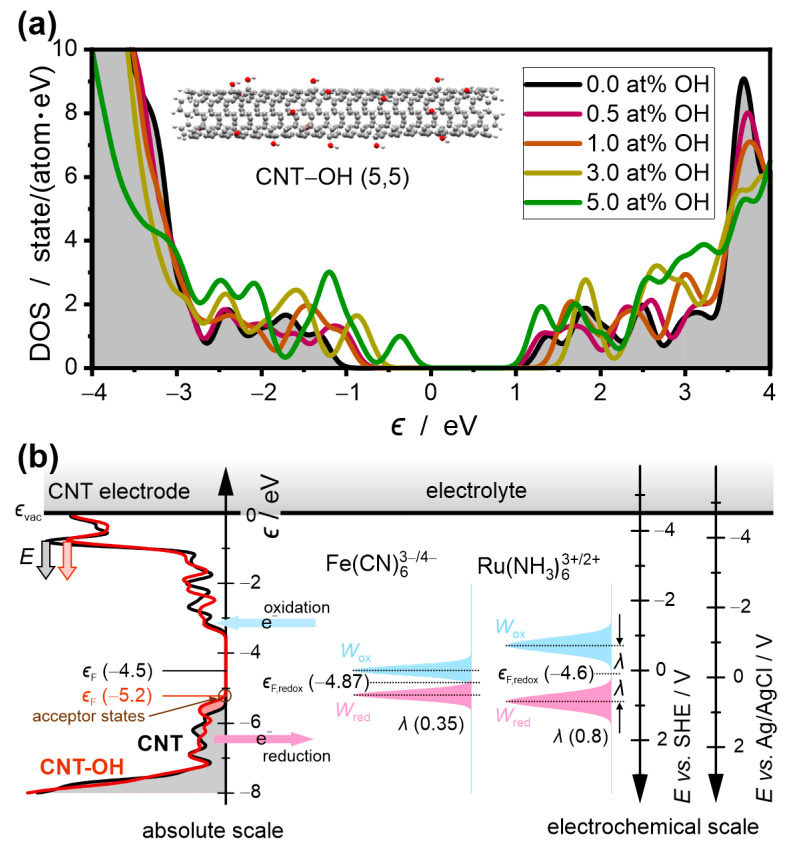
(**a**) The DOS of the hydroxylated CNT(5,5) calculated using DFT. The concentration of the covalently bonded -OH groups changes from 0 to 5 at%. Zero in energy scale corresponds to the $\epsilon_{F}$. of the pristine (5,5). (**b**) The energy level diagram for HET from CNT and CNT-OH (0.5 at%) electrodes to Fe(CN)_6_^3−/4−^ and Ru(NH_3_)_6_^3+/2^ redox couples. The values of the Fermi energy of the CNT electrodes, $\epsilon_{F}$, and the redox couples, $\epsilon_{F,redox}$, are displayed in brackets in eV in the absolute energy scale in which the vacuum level, $\epsilon_{vac}$, is the reference point. The increase in the electrical potential, *E*, of the CNT electrode causes the DOS to shift downward on the energy scale. On the axes to the right of the diagram, the absolute energy scale is converted to the electrochemical scale. The transition from the absolute energy scale (in eV) to the electrochemical scale (in V) is made according to the following formulas [43]: eE (vs. SHE)=−4.5 eV−ϵ and eE (vs. Ag/AgCl)=−4.697 eV−ϵ.

The results of the DOS calculation using DFT are shown in Figure 5a. The DOS of the pristine nanotube above and below the Fermi level shows peaks that reflect van Hove singularities separated by a *quasi*-band gap with a width of approximately 2 eV, characterizing an extremely low DOS. With an increasing concentration of -OH groups, new electron states below the Fermi level are generated. The distribution of these states changes significantly with the concentration of -OH groups. In the case of an extremely high concentration of -OH groups of 5at%, the huge maximum of these states is located at approximately −1.2 eV, which is accompanied by a slightly weaker broad feature with a maximum at −0.4 eV. These -OH-induced states play a pivotal role in the hole doping of CNTs, which translates into electrocatalysis.

Hydroxylation has a much less effect on a DOS above the Fermi level. At low concentrations of hydroxyl groups (0.5 at%), the maxima slightly shifted to lower energy. At the higher concentrations of -OH (1 and 3 at%), the states forming the conduction band shift to the higher energies. However, at the highest considered concentration of the hydroxyl groups (5 at%), the bottom of the conduction band shifts back down to a level slightly lower than in the pristine CNT.

### 3.6. The Alignment of the Electron States of the Redox Couples and the CNT-OH Electrode

According to the Gerischer model on HET at the electrode–electrolyte interface [43,44,45,46], the direction and rate of HET between the electrode and redox species are determined by the energy level alignment between the $\epsilon_{F}$ of the CNT electrode and electrochemical potential, $\epsilon_{F,redox}$, of the redox couples as shown in Figure 4b. The diagram assumes that, in pristine CNTs, the $\epsilon_{F}=-4.5\;eV$. The position of the Fermi level in the absolute energy scale is an averaged value derived from the work function of various nanotubes [47]. The standard potentials of Fe(CN)_6_^3−/4−^ and Ru(NH_3_)_6_^3+/2+^ are +0.37 V and +0.1 V vs. SHE, respectively [48]. After conversion to the energy scale, the equivalent $\epsilon_{F,redox}$ of the Fe(CN)_6_^3−/4−^ and Ru(NH_3_)_6_^3+/2+^ redox species in the solution was set at −4.87 eV and −4.6 eV. The empty oxidized states $W_{ox}$ and the occupied reduced states $W_{red}$ of the redox couples are distributed above and below $\epsilon_{F,redox}$. The centers of the Gaussian distribution of the redox states are separated by the doubled reorganization energy λ*,* where λ=0.35 eV and 0.8 eV for Fe(CN)_6_^3−/4−^ and Ru(NH_3_)_6_^3+/2+^, respectively [49].

The rate of the anodic reaction is proportional to the density overlap between the empty states of CNT and occupied reduced states of the redox couples at specified electrode potential E. The HET rate of oxidation is proportional to $k_{a}\left(eE\right)\propto \int_{-\infty }^{\infty }[1-f(\epsilon -eE)]\;DOS(\epsilon -eE){\;W}_{red}(\epsilon )\;d\epsilon$, where f(ϵ) is the Fermi–Dirac distribution. Since the maximum of $W_{red}$ of Fe(CN)_6_^3−/4−^ at the zero electrode overpotential is located closer to the bottom of the CB of CNT (approximately −2.2 eV) than reduced states of Ru(NH_3_)_6_^3+/2+^ (approximately −2.4 eV), the oxidation of the Fe(CN)_6_^4−^ is slightly more favored than the oxidation of Ru(NH_3_)_6_^2+^. Therefore, it cannot be considered as the main factor responsible for boosting the redox reaction of Fe(CN)_6_^3−/4−^.

### 3.7. The Acceptor States in CNT-OH

The pivotal role in HET from the redox systems to the CNT electrode is played by acceptor states. They are generated in hydroxylated nanotubes due to the additional states that appear within the *quasi*-band gap of the CNT and the downshift of the $\epsilon_{F}$. Using the dependence $∆\epsilon_{F}={-v}_{F}\hbar \sqrt{{\pi n}_{h}}$, obtained for the graphene in ref. [50], the Fermi velocity of $v_{F}=-1.1\times 10^{8}\;cm\cdot s^{-1}$ and $n_{h}=2.9\times 10^{13}\;{cm}^{-2}$ derived from the Raman data, we determined the shift of the Fermi level by −0.7 eV. The tail of the band formed by the hydroxylation-induced states extends far above the $\epsilon_{F}=-5.2\;eV$. The acceptor states in the tail of the DOS give the major contribution to the increased activity toward oxidation of the redox system. The oxidation reaction rate is higher, and the better position of $W_{red}$ of the redox system is matched to the position of the acceptor states.

## 4. Discussion

In this work, we have shown that the hydroxylated CNTs can be considered an efficient hole oxidation system. For this purpose, we have examined binder-free buckypapers consisting of CNT-OHs and pristine CNTs (as a reference). The bulk electrical conductivity of the CNT-OH films was almost five times higher than that of the CNT films, proving the much higher concentration of the free charge carriers in CNT-OH. Due to the hole doping ($n_{h}=2.9\times 10^{13}\;{cm}^{-2}$), the standard rate constant of HET between CNT-OH and the Fe(CN)_6_^3−/4−^ redox couple ($25.9\times 10^{-4}\;cm\cdot s^{-1}$) is more than four times higher than between pristine CNTs and Fe(CN)_6_^3−/4−^ ($6.3\times 10^{-4}\;cm\cdot s^{-1}$). The opposite result is obtained for the Ru(NH_3_)_6_^3+/2+^ redox probe. The $k_{0}=1.4\times 10^{-4}\;cm\cdot s^{-1}$ obtained on the pristine CNT electrode markedly decreased to the value of less than $0.1\times 10^{-4}\;cm\cdot s^{-1}$ at the hole-doped CNT-OH. The quenching of HET from Ru(NH_3_)_6_^2+^ to OH-CNT is caused by the positive charge of the reduced form, which resulted in the limited access of the Ru(NH_3_)_6_^2+^ ions to hole-doped nanotubes. Another factor that influences the oxidation rate is the alignment of the occupied reduced states of the redox system and the acceptor states generated above the $\epsilon_{F}$ of the CNT-OH electrode.

Thus, we have shown that CNT-OH can be an efficient hole oxidation system if the following two conditions are met: (1) The charge of the oxidized ions is negative, and (2) the position of the occupied states of the redox couple on the energy scale is matched to the position of the acceptor states of CNT-OH. As the DFT calculations show, we can tune the position of acceptor states on the energy scale by changing the concentration of the -OH groups covalently bound to the nanotubes. We can easily monitor the concentration of the holes using Raman spectroscopy. Our findings can be used in the construction of selective electrochemical sensors based on CNT-OH electrodes, which can be tuned to a specific analyte. This is especially true for analytes of high physiological importance, which exhibit poor kinetics of electrochemical oxidation reactions. Here, we can mention cysteine, glutathione, glucose, dopamine, ascorbic acid, and uric acid, among others.

The proposed methodology opens the route for investigation of other hole oxidation systems, which can be obtained by functionalization of CNTs with, for example, -NH_2_, -COOH, and -SH groups. These may contribute to the development of novel electrode materials for sensors and to tune its properties to specific analytes.

## Figures and Tables

**Table 1:** The comparison of the position and the full width at half maximum (FWHM) of the Raman G-band and relative intensities of the D and 2D bands in the MWCNT and MWCNT-OH buckypapers.

Buckypaper	$\omega_{G}/{cm}^{-1}$	${FWHM}_{G}/{cm}^{-1}$	$I_{D}/I_{G}$	$I_{2D}/I_{G}$
MWCNT	1575.6	14.4	0.135	1.18
MWCNT-OH	1578.3	27.3	1.188	0.65

**Table 2:** The comparison of the anodic charge transfer coefficient, $\alpha_{a}$, and standard rate constants of HET between redox probes and buckypaper electrodes, $k_{0}$.

Buckypaper Electrode	${Fe(CN)}_{6}^{3-/4-}$		$Ru(NH_{3}{)}_{6}^{3+/2+}$	
	$\alpha_{a}$	$k_{0}[\times 10^{-4}cm\cdot s^{-1}]$	$\alpha_{a}$	$k_{0}[\times 10^{-4}cm\cdot s^{-1}]$
MWCNT	0.493	6.3	0.539	1.4
MWCNT-OH	0.517	25.9	0.738	<0.1

## Data Availability

The original contributions presented in the study are included in the article, further inquiries can be directed to the corresponding author.
